# Supplementary material for: Infection dynamics of COVID-19 virus under lockdown and reopening
Source: Sci Rep. 2022 Jan 27;12:1526. doi: 10.1038/s41598-022-05333-5 (PMC8795434; doi:10.1038/s41598-022-05333-5)
Supplement: Supplementary file 1 — Supplementary Figures. [file 41598_2022_5333_MOESM1_ESM.pdf]

Supplementary Information: Infection dynamics of COVID-19 virus under lockdown and reopening

Jakub Svoboda[a], **Josef Tkadlec**[b], **Andreas Pavlogiannis**[c], **Krishnendu Chatterjee**[a], **and Martin A. Nowak**[b,d,*]

[a]IST Austria, Klosterneuburg, A-3400, Austria
[b]Harvard University, Department of Mathematics, Cambridge, MA 02138, USA
[c]Aarhus University, Department of Computer Science, 8200 Aarhus, Denmark
[d]Harvard University, Department of Organismic and Evolutionary Biology, Cambridge, MA 02138, USA
[*]martin_nowak@harvard.edu

ABSTRACT

This is a Supplementary Information for the manuscript "Infection dynamics of COVID-19 virus under lockdown and reopening". It includes three additional figures.

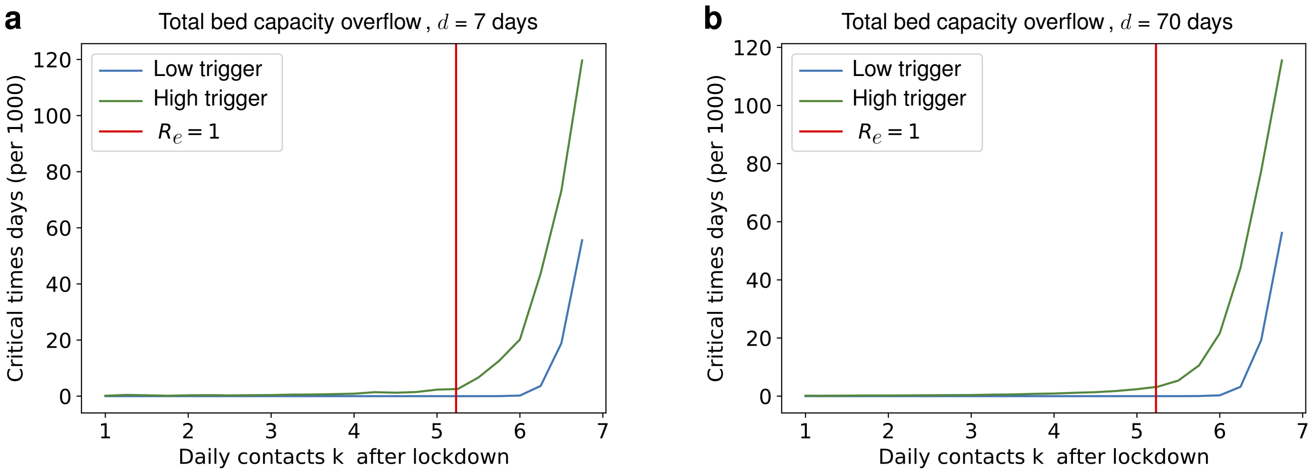

Supplementary Figure 1. Another performance measure: The total (expected) overflow of the bed capacity, per 1000 individuals. For a given stochastic run, the total bed capacity overflow is defined as $\sum_{t \geq 0} \max\{C(t) - c, 0\}$, where C_t is the number of critical cases on day t. The parameters are as in Fig. 3 from the main text ($\tau_{\text{low}} = 3$, $\tau_{\text{high}} = 12$, $k^\star \doteq 5.3$, the patience parameter is **a,** $d = 7$ days and **b,** $d = 70$ days). We note that for high-trigger policies and $k < k^\star$, the total bed capacity overflow is negligible, even though the overflow probability p_{fail} is substantial (see Fig. 3b from the main text).

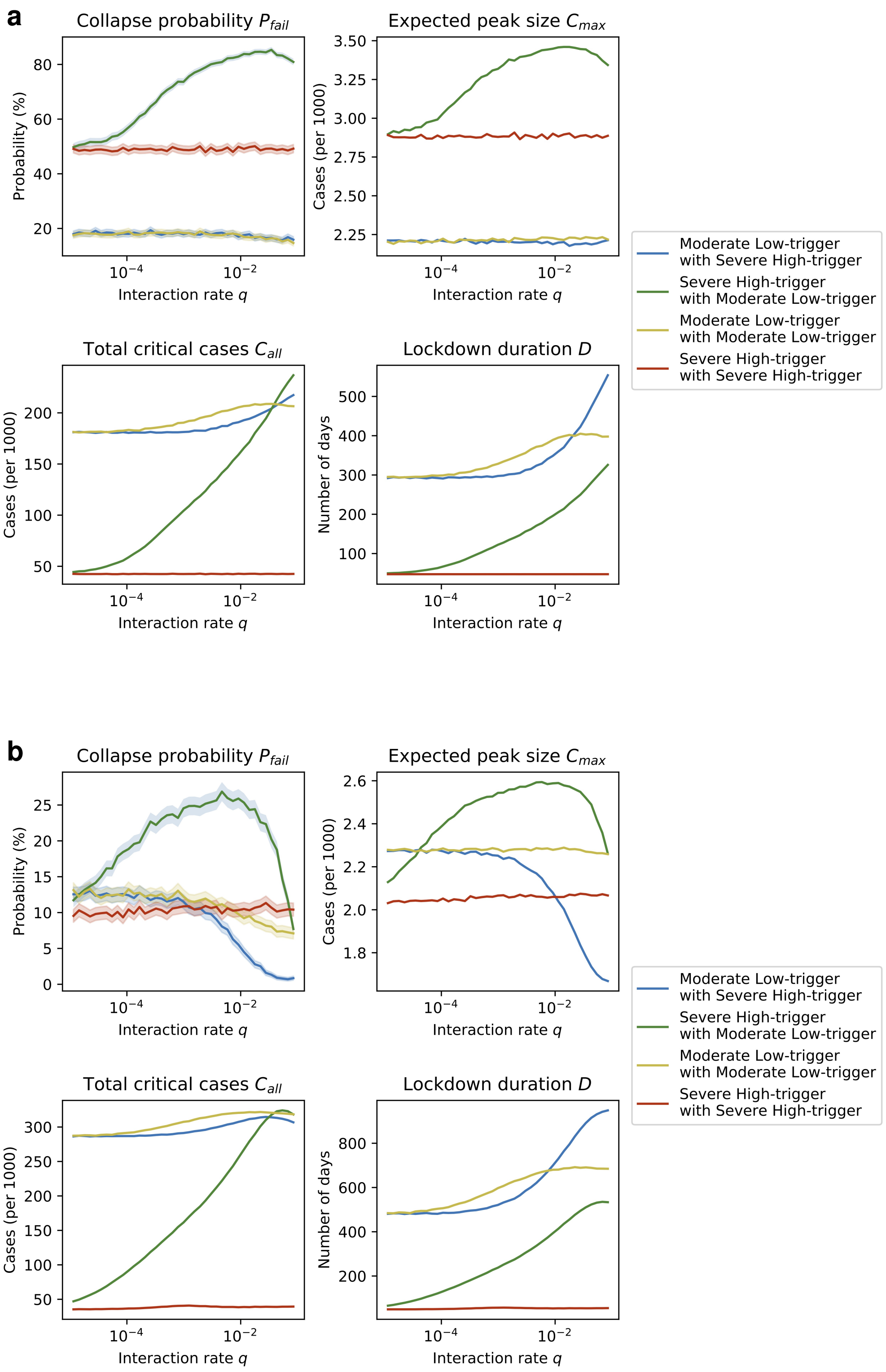

Supplementary Figure 2. Different parameter choices. We perform the computation leading to Fig. 4 from the main text for different parameter choices. In order to keep the reproductive ratio roughly fixed, we always vary two parameters at a time. **a,** We consider a population with $2\times$ shorter infectious period $X_{I\to R} = 5$ days, and roughly $2\times$ larger individual transmission rate $p = 3.6\%$. **b,** We consider a population with $3\times$ as many contacts $k_0 = 45$ and $3\times$ smaller individual transmission rate $p = 0.67\%$. In both cases, the trends in the resulting plots are qualitatively the same as in Fig. 4 from the main text.

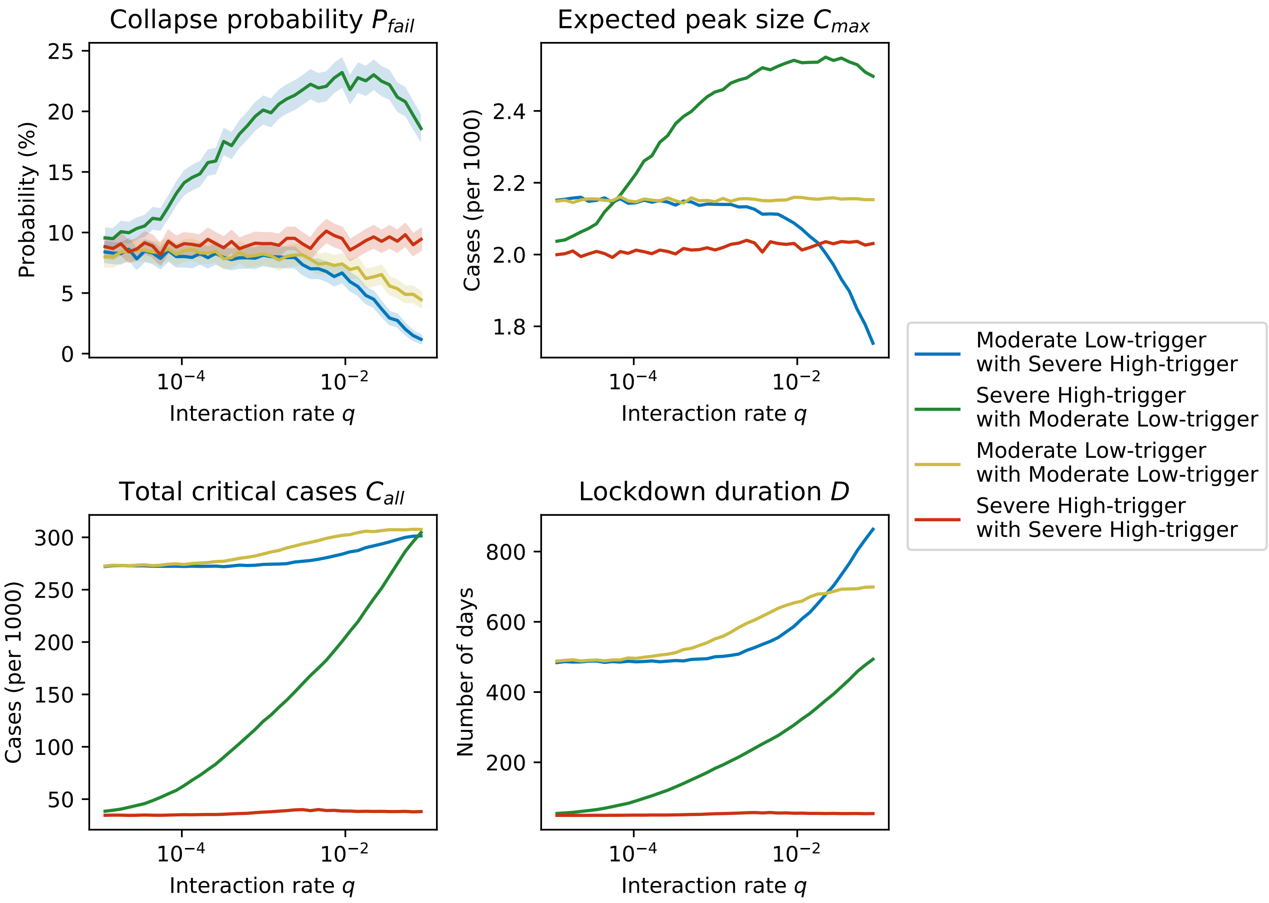

Supplementary Figure 3. Periods following a distribution. We consider a setting in which the random variables corresponding to the pre-infectious period $X_{E\to I}$, the infectious period $X_{I\to R}$, and the critical period $X_{C\to R}$ each follow a distribution over 3 consecutive integer values, rather than being concentrated on a single value. In particular, each variable attains its expectation with probability 50% and is 1 lower or 1 higher with probability 25% each. The resulting plots are qualitatively the same as in Fig. 4 from the main text.
